# Opinions of Youngsters with Congenital Below-Elbow Deficiency, and Those of Their Parents and Professionals Concerning Prosthetic Use and Rehabilitation Treatment

**DOI:** 10.1371/journal.pone.0067101

**Published:** 2013-06-24

**Authors:** Ecaterina Vasluian, Ingrid G. M. de Jong, Wim G. M. Janssen, Margriet J. Poelma, Iris van Wijk, Heleen A. Reinders-Messelink, Corry K. van der Sluis

**Affiliations:** 1 Department of Rehabilitation Medicine, University Medical Center Groningen, University of Groningen, Groningen, The Netherlands; 2 Department of Rehabilitation Medicine, Erasmus Medical Center Rotterdam, Rotterdam, The Netherlands; 3 Rehabilitation Center De Sint Maartenskliniek, Nijmegen, The Netherlands; 4 Rehabilitation Center De Hoogstraat, Utrecht, The Netherlands; Edinburgh University, United Kingdom

## Abstract

**Background:**

Youngsters with unilateral congenital below-elbow deficiency (UCBED) seem to function well with or without a prosthesis. Reasons for rejecting prostheses have been reported earlier, but unfortunately not those of the children themselves. Furthermore, reasons for acceptance are underexplored in the literature.

**Objectives:**

To investigate opinions of children and early and late adolescents with UCBED, and those of their parents and healthcare professionals, concerning (1) reasons to wear or not to wear prostheses and (2) about rehabilitation care.

**Methods:**

During one week of online focus group interviews, 42 children of 8–12 y/o, early and late adolescents of 13–16 and 17–20 y/o, 17 parents, and 19 healthcare professionals provided their opinions on various topics. This study addresses prosthetic use or non-use of prosthetics and rehabilitation care. Data were analyzed using the framework approach.

**Results:**

Cosmesis was considered to be the prime factor for choosing and wearing a prosthesis, since this was deemed especially useful in avoiding stares from others. Although participants functioned well without prostheses, they agreed that it was an adjuvant in daily-life activities and sports. Weight and limited functionality constituted rejection reasons for a prosthesis. Children and adolescents who had accepted that they were different no longer needed the prosthesis to avoid being stared at. The majority of participants highly valued the peer-to-peer contact provided by the healthcare professionals.

**Conclusions:**

For children and adolescents with UCBED, prostheses appeared particularly important for social integration, but much less so for functionality. Peer-to-peer contact seemed to provide support during the process of achieving social integration and should be embedded in the healthcare process.

## Introduction

Congenital upper limb defects affect between 19.5 and 21.5 births per 10,000 [1], [2]. A considerable group of congenital upper limb anomalies result in reduction deficiencies (5.56 births per 10,000) [3]. Children with such impairments often receive prosthetic treatment in order to improve their functionality and to avoid developmental problems [4]. It is doubtful that prostheses fulfill these aims, since the rejection rate is high 35–45% [5], while no difference in functionality is seen between prostheses wearers and non-wearers [6], [7]. Furthermore, prosthesis use seems to reduce manipulation, exploration, variation, and adaptation in the daily-life activities of young children with unilateral congenital below-elbow deficiency (UCBED) [8]. By developing compensatory strategies and auxiliary movements using other body parts (e.g., head, legs, and trunk) to perform a task [9], children also tend to be more independent without prostheses [4]. Thus it is still unclear why some continue wearing prostheses.

Prostheses are typically accepted when people with upper limb impairment face a great deal of difficulty in daily-life activities, have a higher level of amputation (above the elbow), when the abilities of the prostheses are considered to be “fair,” and when wearers are satisfied, in general, with their healthcare [10]–[12]. Advantages of early fitting with a prosthesis in children with UCBED are inconclusive in the literature [13]–[15] and are not associated with satisfaction with the prosthesis, functional use of the prosthesis, or motor skills [16].

Prostheses are often rejected when people do not experience many challenges in daily-life activities, have lower levels of amputation, are unsatisfied with certain features of the prostheses (sweating, cosmesis, or interface discomfort), or are unsatisfied with all healthcare areas (i.e., fitting, follow-up, repair, training, and information provision) [10]–[12]. Abnormal truncal movements that usually accompany the performance of activities in prosthetic users may also determine the rejection of prostheses [17]. Parents also play a role in the rejection of prostheses mostly because of disappointment with the limited benefits of prostheses, insufficient involvement in the treatment, and disappointment regarding socio-emotional guidance [13].

The literature is generally concerned with the reasons for rejection of prostheses in adults and provides abundant information as to quantitative outcomes. Information on self-reported reasons that elucidate why children and early and late adolescents choose or continue to wear a prosthesis is scarce. Knowing how psychosocial factors, vis-à-vis the more technical aspects, contribute to the rejection or acceptance of the prosthesis would be of great interest. Children’s and adolescents’ ideas about what aspects could be improved in a prosthesis have yet to be investigated. The rationale or role of the parents in choosing a prosthesis or in the decision to wear one is also unclear. The approach healthcare professionals take toward improving children’s quality of life, including prosthetic prescription, has been previously described [18]–[20]. Nevertheless, there is not much information about patients’ feedback about rehabilitation care, especially the feedback from children. Therefore, the direction of the current study is aimed at elucidating these aspects of how youngsters with UCBED function; the means chosen is a qualitative study design.

The aims of this study are (1) to investigate the opinions of children and early and late adolescents with UCBED, and that of their parents and professionals as to the reasons to wear or not to wear prostheses, and their opinions about (2) rehabilitation care, and to compare the differences in opinions and perspectives among children, early and late adolescents, parents, and healthcare professionals.

## Methods

The current study is a part of a larger study which focused on the aspects of functioning of children and adolescents with UCBED: activities, participation, prosthetic use or non-use, psychosocial functioning, and rehabilitation care. The results concerning activities and participation, and those concerning psychosocial functioning have been published by De Jong and colleagues [21], [22]. The aim of this published first study was to assess whether youngsters with UCBED encounter activity or participation limitations and, if so, what are their coping strategies for those limitations. The published second study investigated the psychosocial functioning of youngsters with UCBED, with a focus on their feelings about their deficiency and what their coping strategies are in terms of those feelings. The larger study as a whole was designed as a qualitative research study, using online focus group interviews for the data collection.

### 1 Study Design

Qualitative studies offer the possibility of gaining insight into underexplored research topics. Online focus group interviews are useful for exploring opinions, for obtaining a range of views from different age categories, and for observing interactions among a wide range of participants. Compared to classic face-to-face focus groups, the online version offers anonymous participation which minimizes the influence of social pressure and favors a more open interaction; it provides a comfortable environment, and by avoiding the transcription process is inexpensive and time-efficient [23]–[25]. Online focus group interviews were considered appropriate for this study, because they are specifically suitable when rare diseases are the subject of interest and participants live in a widespread area. A group of 8 to 15 participants is believed to work successfully in asynchronous focus groups [26]–[28], and even 19 participants have been used in online settings [29].

### 2 Ethics Statement

Ethical approval was obtained from the Medical Ethical Committee of the University Medical Center Groningen, the Netherlands (number M09.079327). Each participant or child’s parent/guardian provided an informed consent, and completed a demographic questionnaire prior to the beginning of the study. For the participation of the youngsters in the study, informed consent was obtained from the parents/guardians of those participants aged 8–11 y/o, from the participants aged 12–17 y/o and also from their parents/guardians, and it was obtained from the participants only, when aged 18–20 y/o. Regardless of whether their child actually participated, parents/guardians signed a separate informed consent allowing their own participation in the focus group interviews.

The participants were informed that they could contact an independent physician for any distress they experienced and that they could withdraw from the study at any time without any consequences. The confidentiality of the participant was also ensured by assigning a codename (for example, children were given names of types of fruit) to every participant. These codenames were used by the participants during the study and for the purpose of analysis. The credentials of the participants were accessible only to the researchers and to no one else.

### 3 Population

Five categories of participants were considered: children, early and late adolescents, parents, and healthcare professionals who had worked with the UCBED population.

#### 3.1 Inclusion criteria for children and early and late adolescents

Purposive sampling was used [30], meaning that both prosthetic wearers and non-wearers with particular characteristics were selected: (1) aged between 8 and 20 years old, and (2) UCBED at a transradial level with a non-syndromic cause. Three categories were defined in concordance with school age: children aged 8–12 years old (primary school), early adolescents aged 13–16 years old (secondary school), and late adolescents aged 17–20 years old (secondary or higher education). By grouping participants in age categories, we aimed to detect specific age-related opinions on the research topics.

#### 3.2 Inclusion criteria for parents and healthcare professionals

Eligible parents were those whose children met the criteria of inclusion for children and early and late adolescents. Eligible healthcare professionals were those with work experience with the UCBED pediatric group.

#### 3.3 Exclusion criteria

Individuals with insufficient proficiency in the Dutch language and limited mental capacity were excluded.

#### 3.4 Recruitment

Participants (except for healthcare professionals) were recruited through national rehabilitation centers and patient organizations. Patient organizations advertized the study on their websites and in newsletters. Twenty-five random people per group were approached, taking into account age, gender, prosthetic wearing/non-wearing, and referral center. Participants received a package with detailed information, a form for informed consent, and a letter approved by the attending rehabilitation physician stating that the physician supports the study and inviting the child or the parent to participate. Professionals were approached through rehabilitation centers and orthopedic workshops in the Netherlands.

### 4 Procedure

An expert provided methodological recommendations for designing and conducting the online focus group interviews. A website with five forums, one forum per group, was designed to facilitate the online focus group interviews. Participants were able to log in anonymously and post messages at any time of the day they preferred and from the location they preferred, within the timeframe of one week. Participants were instructed to omit names of people or rehabilitation centers.

A question about a specific topic was posted every morning during the first five days. The last two days were assigned to open discussions between group participants. The participants who did not access the website on a particular day would receive a reminder the following day asking them to answer not only the current day’s question but also the question from the previous day. The participants were required to post at least one message as an answer to each of the five questions.

This study addressed aspects of the prosthetic use or non-use (day 3) and rehabilitation care (day 5), formulating queries as follows: “Tell us why you wear or do not wear a prosthesis,” “Tell us how you evaluate the rehabilitation team and technicians,” and “Do you have suggestions for improvement for them?” The rest of the topics were covered on other days: activities (day 1), participation (day 2), and psychosocial functioning (day 4).To ensure the correct understanding of the questions, the authors formulated them according to the participant’s age. The study questions and the website with its five forums were pilot-tested on a group of non-impaired children and independent adults. Minor difficulties with understanding the questions and with using the forums were encountered during the pilot test. The website and the questions were improved based on participants’ suggestions. To enable the comparison of perspectives between groups, parents and professionals were asked to express their feedback from the child’s perspective. Multiple perspectives are important for gaining a richer and broader understanding of the studied population [27], and to help clinicians find suitable solutions for the barriers experienced by the parties dealing with UCBED, that is, children, early and late adolescents, parents, and healthcare professionals.

In order to cover a broad area of interest, the questions were based on the World Health Organization’s International Classification of Functioning, Disability and Health for Children and Youth (ICF-CY). ICF-CY addresses issues on two levels: functioning and disability (body functions, body structures, activities, and participation), and contextual factors (environmental and personal factors) [31].

In order to address a possible bias induced by the lack of nonverbal communication, emoticons were made available. This enabled participants to express their feelings. Two moderators were online every day of the study from 8 a.m. to 11 p.m. to ensure that the online focus group interviews were conducted properly. They (IdJ and HRM) followed the moderator’s principles [32] to allay some of the moderator’s influences. The two moderators facilitated an interactive discussion between participants, but avoided influencing or dominating the discussions. Moderators refrained from rephrasing and evaluating statements; instead, they repeated comments using the participant’s words, and provided positive reinforcement by using neutral comments and probing. Both moderators were experienced in the field of child and hand rehabilitation, in addition to a background in human movement sciences, and were not involved in the treatment of the participants. HRM had experience with qualitative data collection methods in pediatric populations. Moderators were in contact, during the study period, with a very experienced rehabilitation physician working with this type of patient. Whenever clarifications of an answer were needed or new information/issues appeared, moderators posted additional questions to individual or all participants until no other new information appeared. This is similar to reaching data saturation [30]. All the data is available in the Dutch language or, if requested, a translation in English can be provided as well.

### 5 Data Analysis

The most common methods in healthcare research used to analyze qualitative data are thematic analysis, grounded theory, and the framework approach [33]. The framework approach enables, as does thematic analysis, the corroboration of predefined research questions with the themes that emerge in the study. The advantage, however, is that it starts deductively from the clearly predefined objectives of the study, and is systematic and transparent, allowing easy access to the analytical process for the researcher as well as for other people [34]. The framework approach was used to analyze the data from this study. The approach contains five steps in which data is screened, condensed, and mapped into a thematic framework:

#### 5.1 Familiarization

The data generated on the days allocated to prosthetic use and rehabilitation care were read by three authors (EV, HRM, and CvdS). The rest of the data was also read to extract remarks about prosthetic use and rehabilitation care. Key ideas and themes were identified in a meeting with the three authors. The themes were derived from subjects frequently mentioned by the participants.

#### 5.2 Identifying a thematic framework

A coding framework was developed by EV to structure the collected information around key issues and themes (Table S1). Based on the aims of the study, the themes were grouped into main categories such as “reasons to wear a prosthesis,” “reasons not to wear a prosthesis,” or “tips for making a prosthesis better, adaptive devices, and other creative solutions.” In addition, for each main category a “general” theme category was created for data not matching the other themes. The data in the “general” theme category (e.g., frequency, time and place for wearing the prosthesis) when considered appropriate were made available in the Results section to provide detailed information for the themes.

#### 5.3 Indexing

EV and HRM tested the coding framework on ten percent of the data. After discussing minor differences in the manner of coding, agreement was reached upon the final version of the coding framework. EV correlated text pieces from the entire dataset with the appropriate code.

#### 5.4 Charting

EV displayed the pieces of text corresponding to the matched code and affiliation group in the form of a matrix. The columns contained the framework themes, while the lines contained each participant’s quotes on the theme. The quotes of wearers or non-wearers were thus easily identifiable from the matrix. The data accessibility of the matrix facilitated the analysis of the perspectives of the different groups, and of wearers and non-wearers.

#### 5.5 Mapping and interpretation

The resulting matrix was verified for the correct code by HRM and CvdS. In order to draw conclusions, EV, HRM, and CvdS analyzed the matrix separately. All three discussed the similarities and differences that occurred. Consensus was found on interpretations and conclusions.

## Results

From the total of 125 eligible participants, 77 (62%) participated in the study. Forty-two were either children, early adolescents, or late adolescents; 16 were parents; and 19 were healthcare professionals. No differences in age, gender, and provenance center were found between participants and non-participants. Non-wearers were represented by participants who had experience with prostheses (children 47%, early adolescents 54%, late adolescents 58%, and children of parents 63%), and participants without previous prosthetic experience (Table 1). Myoelectric prostheses were the most popular among wearers (Table 1). The healthcare professionals group consisted of five physiatrists, six occupational and physical therapists, six certified prosthetists, and two psychologists.

**Table 1:** Characteristics of participants (n = 77).

Characteristics	Children	Early Adolescents	Late Adolescents	Parents
	No.(%) or Minimum-Maximum	No.(%) or Minimum-Maximum	No.(%) or Minimum-Maximum	No.(%) or Minimum-Maximum
Participants (approached, recruited, participated)	25, 17, 17	25, 15, 13	25, 13, 12	25, 19, 16
Distribution^a^	3, 3, 4, 4, 3	2, 3, 3, 5, 0	2, 3, 4, 3, 0	3, 3, 4, 6, 3
Gender (Male/Female)	9/8 (53/47)	3/10 (23/77)	4/8 (33/67)	10/6 (62.5/37.5)^b^
Age	8–12	13–16	17–20	12^b^
Age of fitting first prosthesis	9 mos.-8 y/o	6 mos.-8 y/o	6 mos.-9 y/o	6 mos.-6 y/o
User status				
Wearer	2 (12)	6 (46)	5 (42)	1 (6)^b^
Wearing frequency of current prosthesis (hours per day)	7.5, 4^c^	1–14	1.5–12	12^c^
Non-wearer	15 (88)	7 (54)	7 (58)	15 (94)^b^
Never wore prosthesis	7 (41)	–	–	5 (31)^b^
Type of current prosthesis	2 (12)	6 (46)	5 (42)	1 (6)^b^
Without grip function	–	1 (8)	2 (17)	–
With grip function	2 (12)	6 (46)	5 (42)	1 (6)^b^
Body powered	–	2 (15)	–	1 (6)^b^
Myoelectric	2 (12)	3 (23)	3 (25)	–
Type of prosthesis at first fitting	10 (59)	13 (100)	12 (100)	11 (69)^b^
Without grip function	5 (29)	12 (92)	9 (75)	6 (38)^b^
With grip function				
Body powered	1 (6)	–	1 (8)	3 (19)^b^
Myoelectric	4 (24)	–	1 (8)	2 (13)^b^
Unknown	–	1 (8)	1 (8)	–

Notations: mos. = months, y/o = years old.

a Number of participants distributed per participating rehabilitation center; the last number represents the number of participants recruited through other centers/organizations.

b Characteristics of children of participating parents.

c The values represent the actual number of hours per day (two wearers in children group and one wearer in parents’ group).

The participants were active in interacting with each other and with moderators. Each participant posted at least one message as an answer to each study question. Parents and healthcare professionals provided the most extensive answers.

### 1 Reasons to Choose and Wear Prostheses

#### 1.1 Cosmetic, social, emotional, and identity reasons

Prostheses were chosen and worn primarily to provide cosmesis. Cosmesis helped participants of all age categories to manage relationships with the people in their environment. A frequently mentioned reason was to prevent adverse reactions like teasing and staring. For children, the prosthesis also offered a normal body appearance, while for early and late adolescents wearing a prosthesis allowed them to establish a good first impression and gave them a feeling of self-confidence.

“For walking on the street I found it [the prosthesis] enjoyable; everyone finds you normal then, because you then have two hands.” (10 y/o girl, non-wearer with prosthesis experience)

The prostheses were worn every day, yet limited to being worn in public. In a safe home environment, the prosthesis had nothing to add and was therefore removed. The cosmesis also became more important during transitional periods such as puberty.

“At puberty, I noticed that they ask for it [the prosthesis] from a cosmetic point of view… They especially want a prosthesis, for example, when they go to secondary school.” (Healthcare professional)

Professionals noticed that rejection of prosthesis use occurred in some children as soon as they became accustomed to a new environment.

#### 1.2 Functionality, manipulation, dexterity reasons

Along with cosmesis, functionality was important for children and adolescents in the process of choosing and wearing prostheses. Being able to experience activities of daily life in a normal way, to grip with the impaired upper limb, and curiosity about whether the prosthesis offered more dexterity also led participants to opt for prostheses.

“A cosmetic prosthesis often has to be practical too; that’s why children/adolescents often want a myo [myoelectric prosthesis] then.” (Healthcare professional)

“I wanted to know if it would be handy or not to wear a prosthesis. I wanted to try and become handier so that everything might be a bit easier. ” (13 y/o girl, non-wearer with prosthetic experience)

Wearers and non-wearers regarded the prosthesis as a “useful help accessory” for activities like managing school tasks, cutting, grasping, holding, and lifting.

Activity-specific use was noticed in early and late adolescents for activities such as cycling and driving more safely, or for leisure purposes such as playing sports like volleyball, hockey, and football.

At other times, participants managed to function perfectly well without prostheses. However, activities such as lifting heavy objects, playing sports like volleyball or hockey, or doing some jobs such as delivering newspapers were not performed without prostheses by several early adolescents.

#### 1.3 Physical reasons

Some prosthetic wearers in every group considered wearing a prosthesis as something beneficial for muscle development, locomotion, posture, and balance.

“When I play soccer, I have my prosthesis on… I have the feeling that I have better balance with it [the prosthesis] on and that I can manage better if I fall.” (16 y/o girl, wearer)

#### 1.4 Parents and prosthesis choice

Wearers in children’s and late adolescent groups specified that they had been too young to make the choice on their own when the choice was initially made. Parents had therefore played an important role in the process of acquisition and wearing of prostheses.

Some parents had based their choice on the information and instructions about the benefits of early fitting that they had received from healthcare professionals. Other parents had followed their personal beliefs. They wanted to give the child the opportunity to experience a prosthesis so as to provide him/her with the knowledge to be able to make an informed choice later in life. Another reason for parents to choose a prosthesis for their child was that they had wanted to overcome the emotional stress of having a child with an upper-limb impairment.

“When she was little, we allowed our daughter to use a prosthesis in the morning and go without the prosthesis in the afternoon. This way she could discover herself what was most suitable for her.” (Parent of a 13 y/o girl, non-wearer with prosthetic experience)

“There are parents that want a prosthesis per se, because that way they see their child as more complete, and they find it less difficult for themselves and the family.” (Healthcare professional)

### 2 Reasons not to Choose and Wear Prostheses

#### 2.1 Cosmetic, social, emotional, and identity reasons

Child non-wearers confronted the staring issue head on. They wanted acceptance and respect from the environment without having to wear a prosthesis. Early adolescents experienced self-confidence and self-identity without a prosthesis. Professionals explained this self-confidence on the part of adolescents as a result of realizing that they were able to perform everything just as well without the prosthesis.

Late adolescents, non-wearers, had negative feelings regarding the prosthesis. For them, the prosthesis was a statement about being disabled by highlighting the upper limb defect.

“I felt myself disabled with that thing [the prosthesis] on… When I was wearing it, I had the feeling that it even made me stand out more [than without the prosthesis].” (20 y/o girl, non-wearer with prosthetic experience)

Non-wearers with or without prosthetic experience reached the stage of accepting their situation. The prosthesis could not substitute for a real hand; it was “a dead thing” or “a doll’s hand,” and it did not belong to the child. In that sense, the cosmesis of a prosthesis lost its value.

“I did not want it [the prosthesis] anymore and I thought, ‘I am how I am,’ and that worked just as well.” (9 y/o girl, non-wearer with prosthetic experience)

“I never wanted it [the prosthesis] before, because I considered it a fake hand… I’m also not ashamed about it [the affected hand] [smiley face].” (11 y/o boy, non-wearer without prosthetic experience)

#### 2.2 Functionality, manipulation, dexterity reasons

Children and adolescents felt more functional, more dexterous, or faster without prostheses. The majority of non-wearers were able to perform “everything and more” without the prosthesis. Parents and professionals noticed that children and adolescents saw little or no functional value in wearing prostheses.

“Meanwhile he [parent’s child] is at an age now (8 y/o), at which he has become very dexterous with his arm … He doesn’t see his [affected] arm as a limitation and I think for him walking around with a prosthesis the whole day has no added value.” (Parent of an 8y/o boy, non-wearer with prosthetic experience)

Wearers, on the other hand, specified that they did not use their prostheses for activities like eating, playing, tying shoelaces, manual work at school, or working with a computer, because they were more dexterous or had better grip without them.

“I’ve been able to tie my shoelaces with and without a prosthesis since I was 3! I find it easier without the prosthesis, because then I have more grip on the lace.” (15 y/o girl, wearer)

#### 2.3 Technical and interface reasons

The most often mentioned complaint and reason for not wearing the prosthesis was a prosthesis’s weight. The myoelectric prosthesis often required extra support with the sound hand to counterbalance the weight. Discomfort caused by the interface contact with the stump or the technical limitations of the prosthesis itself were also discussed. The interface caused stump irritations, sweating, bad odor, and difficulties fixing the stump in the socket.

“I found it annoying that the prosthesis was just stuck on my arm, and it [the arm] was sweating, and that’s why it [the prosthesis] was difficult at first to put on and off.” (10 y/o girl, non-wearer with prosthetic experience)

The prosthesis had a limited number of movements and grip functions. Other complaints of non-wearers include the presence of liners, frequent technical failure, and damaged or dirty gloves. Putting on and taking off were perceived as a difficult and laborious process. Manufacturing times were considered long, and learning to use a prosthesis was energy- and time-consuming.

Technical issues were not considered by the wearers to be reason enough not to wear prostheses, but rather as aspects that needed improvement.

#### 2.4 Physical reasons

Non-wearers were very disturbed by the lack of sensorial feedback from the stump, along with arm and shoulder fatigue, and pain from using prostheses.

“My arm was really tired after a day wearing a prosthesis and without [the prosthesis] not at all. With the prosthesis on, my shoulder used to start hurting easily. *Were these reasons, a tired arm and pain in the shoulder, the most important reasons to stop wearing the prosthesis?* Yes, actually they were.” (16 y/o girl, non-wearer with prosthetic experience)

#### 2.5 Parents and the prosthesis choice

Parents who did not opt for a prosthesis for their child made this choice because they “first wanted to see his [child’s] functionality without a prosthesis.” Other parents considered a prosthesis to be useless, based on users’ stories about daily-life experiences with prostheses.

### 3 Tips for Improving Prostheses

Late adolescents, parents, and professionals suggested lowering the costs of prostheses. Furthermore, they desired prostheses that were lighter, more attractive, easier to manipulate, and that had more hand positions and separate finger movements, sensorial feedback, and better glove quality. The harnesses on body-powered prostheses seemed to be very annoying, especially for boys:

“Harnesses can indeed be a problem, particularly among boys that want to get rid of the ‘bra’ […].” (Healthcare professional)

#### Alternatives for prosthetic wearing

The participants were creative in developing alternatives to wearing prostheses. The children or their relatives developed special techniques using body parts such as stump, head, trunk, mouth, or knees, and creative strategies such as bandages or tape to tie an object around the stump or to tie a magnet to it for holding objects.

Adaptive devices for the arm or prosthesis received a lot of attention among participants, especially for non-wearers with or without prosthetic experience, and were described as helpful tools for performing specific activities such as cycling, eating, playing sports, and playing a musical instrument. Professionals and parents suggested developing more adaptive devices, although it appeared to be difficult to get the costs of adaptive devices reimbursed.

### 4 Rehabilitation Care

#### 4.1 General opinions

The participants generally experienced good rehabilitation care. Many late adolescents were neutral, perceived the care as appropriate, or could not recall how they had felt about it. The participants had received proper guidance in choosing a prosthesis and had been adequately informed about functioning with a short arm and with a prosthesis.

#### 4.2 Peer contact

A recurrent theme in all groups was peer-to-peer contact. Parents with young children were eager to know what the possibilities and limitations were for their child in terms of normal functioning and development. Parents received answers to these questions during meetings with peer parents. Emotional support from experienced parents diminished the anxiety of less-experienced parents.

“We saw children in the peer-group meetings who were older [than their child] and they told us how they had found a solution for all the little problems. We benefited a lot from this and we still really enjoy going to these meetings… I think it can be very comforting for ‘new’ parents to have contact right away with ‘experienced’ parents so that a lot of the anxiety is taken away.” (Parent of a 13 y/o boy, wearer)

Children and early adolescents also benefitted from peer-to-peer contact. Children referred to those meetings as “fun-time.” Emotional support was offered even during the course of the online focus group to one child who was going through a difficult time.

“Right now I don’t want to be around other children.” (9 y/o boy, non-wearer with prosthetic experience)

Reaction from a participant: “I think it’s sad that ‘codename’ [referring to the previous participant] is so sad; you’ve got to remember that you’re perfect the way you are. [sad face]” (11 y/o boy, non-wearer without prosthetic experience)

Early adolescents added that the meetings were informative and emotionally helpful for them. They found out more about novel prostheses and solutions for performing difficult activities.

“I go about once a year to the meetings. I am the oldest one there, and so many people ask me things. I like this and also learn things, because they [other participants] help you with new things and improvements.” (14 y/o girl, wearer)

The online focus group was seen by the children and early adolescents as an opportunity to share information about ways to perform certain activities like playing a musical instrument, playing sports, or tying shoelaces.

#### 4.3 Psychosocial assistance

Some children regarded the psychologist as vague and found the psychological tests unpleasant, or they simply did not want to talk and answer the question, “How are you doing?” However, early adolescents and parents mentioned that emotional and psychosocial help from the rehabilitation team was useful when they encountered difficult moments.

“I always enjoyed an hour with the social worker the most, always nice talks, and she helped me at the same time with things that were difficult for me at that time, such as bullying and other things.” (16 y/o girl, non-wearer with prosthetic experience)

Professionals all agreed that psychosocial disciplines are an important and valuable part of the rehabilitation treatment.

#### 4.4 Themes discussed by professionals

Professionals recognized that the clients’ expectations were often too high. Children or their parents believed that a prosthesis could solve their problems with the short arm, but the outcome was not always the one they had aimed for.

Although professionals admitted that the current tendency of healthcare providers was to prescribe prostheses, and that more practice was needed until the child performed automatically with the prosthesis, some professionals had different ideas.

“I think that if you consider providing a prosthesis, then you should at least ensure that the child is not clumsier with a prosthesis than without it; so practicing is needed until his prosthesis can be pretty automatically manipulated.” (Healthcare professional)

They stated that the team should not strive for bilateral handling of UCBED children, but that the child should grow up with a positive self-image and should be able to fulfill his wishes with or without a prosthesis. These professionals realized that they should listen carefully to the client’s needs and to the strategies they had already found on their own, and should avoid imposing their own knowledge excessively.

## Discussion

The children and adolescents with UCBED interviewed in our study seemed to choose and wear prostheses mostly for cosmetic reasons in order to avoid people staring at them. In adults with upper limb amputation, similar [35]–[37] and opposite outcomes were found (i.e., cosmesis was less important) [38]. On the other hand, our findings acknowledged that poor prosthetic cosmesis influenced the non-choice and rejection of the prosthesis [10], [13]. The authors of a systematic review noticed a trend in qualitative studies in terms of reporting about the importance of cosmesis [39]. This being the case, the cosmetic aspects of prostheses in youngsters with UCBED deserve the full attention of manufacturers and of those recommending or prescribing them.

In terms of the World Health Organization’s ICF classification, children and adolescents with UCBED have a body structure impairment [31]. Therefore, one might expect their functionality to be affected as well. However, the results of our study suggest that the functionality of children and adolescents is good, since many were able to perform activities with or without prostheses; this idea is supported in the literature as well [6], [7]. The use of creative strategies (using sweatbands and/or other body parts for grasping and holding objects in place, choosing easier activities) to facilitate activities and participation in daily living may be an alternative to the use of prostheses [22].

In contrast to people with acquired arm amputations, children and adolescents with UCBED have no “sense of loss” regarding the short arm [40]. If children and adolescents with UCBED argue that they do not experience activity limitations and participation restrictions and have no “sense of loss,” then there is no reason for them to believe they have an impairment and to feel disabled. However, there are mechanisms that make these youngsters aware of the impairment. Along with body structures and functions, activities and participation, the ICF considers the environmental and the personal factors [31]. Environmental and the personal factors (gender, educational level, ability to adjust) may influence participation of people with amputations [41] and our findings support this.

When the children and adolescents with UCBED in our study did start to use prostheses, people from their close environment (parents, healthcare professionals) or from their external environment (strangers) exerted a great influence in this regard. Providing the child with a prosthesis in order to improve functionality or to disguise the impairment may be considered as strategies on the part of the parents to cope with their child being disabled. These strategies have been previously described [19], [42]. Later on, when children and early and late adolescents become aware of the impact exerted by the short arm on their life, they find solutions to the problems they encounter. In addition to dealing with staring and hostile reactions from people, people with impairment of the upper limb have to deal with their own identity and values concerning body image, sexuality, and career [40]. This is the moment when cosmesis becomes more important and influences the choice of a prosthesis.

In the context of prosthetic use for cosmetic purposes, the concept of normality becomes a matter for discussion. One way to achieve normality for people with disabilities is to adjust and to fit into society [43]. In the research we conducted, participants of all ages experienced a need for normality, especially during transitional periods (a new school or applying for a job), which has been reported in previous studies as stressful events [40], [44], [45]. Therefore, more psychological attention and information about cosmetic options is needed from healthcare providers, especially in critical transitional phases like puberty.

For many children and adolescents in the study, the way to adjust to the environment and to ensure normality was to wear prostheses so as to appear bodily complete. Being able to perform daily, leisure, and school activities in the same way as their non-disabled peers may also be considered a form of normality. In these circumstances, the prosthesis seems to represent a source of empowerment that facilitates integration into society [43]. For a balanced relationship between youngsters with UCBED and their environment, it would also be appropriate for those people in their environment to adjust their way of thinking, perceiving, and approaching youngsters with UCBED.

Another way of achieving normality is to accept and acknowledge the impairment [43]. This was the case with the non-wearers in our current study. The non-wearers’ wish for inclusion in society was based on being valued and accepted as they were. This might well mean that the psychosocial contribution of the prosthesis in combating others’ staring at them is unnecessary after all. By not wearing an unnatural-looking prosthesis, children and adolescents believed they were not altering their appearance. This helped them reinforce their self-esteem and improve their self-identity. In addition, if prostheses are seen as having no functional gain [8], [13], [46], as being technically unsatisfactory and physically uncomfortable [47]–[49], and sometimes actually hampering effective performance [8] – issues we also found in the present research – the added value of the prosthesis disappears and rejection of it occurs. Interestingly, some of the participants succeeded in embracing acceptance and in using the prosthesis for some daily-life activities and in playing sports, a phenomenon also described in the literature [7], [12]. These observations question prosthetic functionality and necessity: “Are prostheses the best solution for children’s and adolescent’s needs?” Our study also highlighted the perceived value children and adolescents expressed regarding the use of adaptive devices. These devices are light-weight, designed for specific activities, easy to manipulate and to put on [50]. Therefore, considering adaptive devices as an option for rehabilitating children and adolescents with UCBED may be of great value.

### Participants’ Perspectives about Prosthetic Use

Study participants, whether wearers or non-wearers, seemed to have the same expectations from a prosthesis when they decided to choose for one (i.e., nicer appearance and better functionality). After wearing and testing it, these expectations were not met for non-wearers, and only partially met for wearers. This discrepancy between a person’s wishes and the outcomes of prosthetic use, detected by healthcare professionals in the present study, has also been reported in the literature by parents of these children [13] and by adults [51]. Providing information and clarifying the real possibilities and limitations of prosthetic use for consumers would serve to balance expectations versus real-life possibilities. More opportunities for trying and using prostheses before purchasing them would allow children and early and late adolescents to make a more informed choice. Providing these opportunities could be organized in the form of banks with prosthetic simulators that could be rented. A prosthetic simulator is a prosthesis which is adapted with fastening systems and can be attached on any type of arm (amputated, normal) [52].

### Rehabilitation Care

The current research results were in line with the findings of other studies that stated that peer-to-peer contact provided emotional assistance for parents and children, as well as understanding, interaction, and identification with people in the same situation [9], [40], [53]. Incorporating regular peer-to-peer meetings into healthcare would address important aspects of the harmonious development of children and early and late adolescents with UCBED.

Patient-centered care was supported by healthcare professionals in our study. Patient-centered care considers three assumptions that would improve rehabilitation care: the patient (1) is the customer, (2) is the “owner of his body, mind, and soul,” and (3) has requested a service in a health matter, so the service provided should focus on the patient’s desires [54].

### Study Strengths and Limitations

A subject of novelty in the literature and a strength of this study is the fact that children, early adolescents and late adolescents themselves were interviewed, and not only people in the immediate environment (e.g., parents), as in the majority of studies. Along with reasons for rejection – preferentially treated in the literature – the current study also explored the determinants for wearing prostheses in children and early and late adolescents with UCBED. Their opinions about prosthetic use and rehabilitation care allowed for a better understanding of the needs that a young person with UCBED experiences at a certain stage of life. The use of online focus group interviews proved to be an efficient method for collecting a large amount of data in a short period of time. For youngsters with UCBED, the online interaction was easy-going and convenient, since it offered anonymity and flexible participation hours [24], [25], [29].

This study also has some limitations. Opinions about prosthetic wear in the children and parents groups may have been underexplored due to the low number of wearers in these two groups. However, in all groups, the majority of the current non-wearers had previously worn prostheses. As such, opinions of non-wearers were also valuable for determining reasons for wearing prostheses. There were more females than males in the early adolescent, late adolescent, and parent groups. They might have influenced the results by highlighting the importance of cosmesis, but studies with a majority of males found the cosmetic aspect very important as well [35], [51], [55]. One may argue that the age of fitting the first prosthesis varies between the groups and might have had an influence on reporting reasons for prosthetic use. No clear proof exists in the literature regarding possible relationships between age of fitting and prosthetic use in later life [15], [16].

The findings of this study should be interpreted in the context of qualitative studies and focus groups. Future studies in larger populations, designed as interviews or questionnaires, might explore in detail the reasons why children and early and late adolescents with UCBED either wear prostheses or do not do so.

### Conclusions

Children and early and late adolescents with UCBED seem to choose and wear prostheses mainly for cosmetic reasons, in order to achieve social integration and not because of limited functionality. Peer-to-peer contact, organized by the rehabilitation teams in conjunction with other institutions, appeared to be an important informational and emotional support for children, early adolescents, and parents. When working with UCBED youngsters there should also be a focus on the importance of the cosmetic possibilities offered by a prosthesis. Extending the treatment options beyond prostheses to other solutions – such as, for example, the use of adaptive devices – would ease some daily-life activities for these children and adolescents. Further research should also focus on the psychosocial events and experiences in this young group.

## Supporting Information

Table S1 **Thematic framework around key issues about prosthetic use.**
(DOC)
