# Supplementary material for: Opinions of Youngsters with Congenital Below-Elbow Deficiency, and Those of Their Parents and Professionals Concerning Prosthetic Use and Rehabilitation Treatment
Source: PLoS One. 2013 Jun 24;8(6):e67101. doi: 10.1371/journal.pone.0067101 (PMC3691115; doi:10.1371/journal.pone.0067101)
Supplement: Table S1 — Thematic framework around key issues about prosthetic use. (DOC) [file pone.0067101.s001.doc]

**Table S1. Thematic framework around key issues about prosthetic use.**

| **Key issues** |
| --- |
| 1. Reasons to choose a prosthesis |
| 1.1 Cosmetic/Emotional/Social/Identity integrity |
| 1.2 Functional/Manipulation/Handling gains |
| 1.3 Parents and prosthesis choice |
| 1.4 Physical improvements (better locomotion, balance, posture, muscle development) |
| 1.5 Technical/Interface advantages (lighter prosthesis) |
| 1.6 Professional advice about the method of choosing (movies, testing prosthesis first, own choice) |
| 1. Reasons not to choose a prosthesis |
| 2.1 Cosmetic/Emotional/Social/Identity inefficiency |
| 2.2 Functionality/Manipulation/Dexterity as non-value |
| 2.3 Parents and prosthesis choice |
| 1. Reasons to wear a prosthesis |
| 3.1 Cosmetic/ Emotional/Social/ Identity integrity |
| 3.2 Functional/Manipulation/Handling gains |
| 3.3 Physical improvements (better locomotion, balance, posture, muscle development) |
| 3.4 Technical/Interface advantages (preference for wearing lighter prostheses) |
| 1. Reasons not to wear a prosthesis |
| 4.1 Cosmetic/Emotional/Social/Identity inefficiency |
| 4.2 Functional/Manipulation/Handling as non-value |
| 4.3 Physical discomfort (no sensitivity, arm or hand fatigue, crooked stance) |
| 4.4 Technical/Interface problems |
| 1. Tips for making a prosthesis better, adaptive devices, and other creative solutions |
| 5.1 Lowering costs |
| 5.2 Technical/aesthetic adjustments |
| 5.3 Adaptive devices and other creative solutions |
| 5.4 More prostheses created especially for children |
